# Aortic Dissection in a Pregnant Patient without Other Risk Factors

**DOI:** 10.1155/2019/1583509

**Published:** 2019-06-20

**Authors:** Priya V. Patel, Raanan Alter, Recia Frenn, Thaddeus P. Waters

**Affiliations:** ^1^Department of Obstetrics and Gynecology, Loyola University Medical Center, Maywood, IL, USA; ^2^Department of Gynecology Oncology, University of Chicago, Chicago, IL, USA

## Abstract

*Background. *An aortic dissection is a life-threatening condition in which the intima of the artery tears causing separation of the intima and media. Pregnancy places women at a significantly increased risk of common vascular events including venous thromboembolism, myocardial infarction, and stroke, while also increasing the risk of rarer vascular events such as aortic dissection and aortic rupture.* Case. * A 30-year-old previously healthy multiparous woman presenting at 36 weeks of pregnancy with a Type A aortic dissection. She underwent a combined emergent cesarean delivery followed by repair of her aortic root.* Conclusions. *Aortic dissection should be high on the differential for pregnant patients presenting with the characteristic complaints and physical exam findings given the high mortality rate associated with this vascular event.* Teaching Points.* (1) This report reviews the characteristic presentation, risk factors, and physical exam findings in a patient with an aortic dissection. (2) The report includes treatment options for pregnant patients based on the classification of the dissection.

## 1. Introduction

An aortic dissection is a life-threatening condition in which the intima, the inner layer of the aorta, tears causing the intima and media, the middle layer, to dissect [1]. There are two main systems for classifying aortic dissections, the DeBakey system and the Stanford classification system. The DeBakey system is an anatomical description of the aortic dissection, categorizing it by where the original intimal tear is located and the extent of the dissection. The Stanford classification is divided into two groups depending on whether the ascending aorta is involved or not. Risk factors for acute aortic syndromes include hypertension, gender, age, family history, connective tissue diseases (i.e., Ehlers-Danlos syndrome and Marfans syndrome), heavy smoking, vascular inflammation, trauma, and pregnancy. The most common presenting symptom is pain, described as severe and sharp, unlike any pain experienced before. The pain occurs in over 90% of patients, with 85% noting the onset of pain to be abrupt and causing the patient to seek medical attention immediately [2]. On physical exam, patients with an aortic dissection may have a pulse deficit, new diastolic murmur, hypotension, or widened pulse pressure. Work-up typically includes 12-lead EKG, troponin, d-dimer, and cardiac imaging. EKG findings typically show nonspecific ST-T wave changes that may mimic myocardial ischemia, thought to be due to an acute decrease in perfusion of the coronary arteries [3]. Troponin levels are often only mildly elevated whereas D-dimer levels are typically markedly elevated compared to the normal elevations expected during pregnancy. Diagnosis is based on imaging with either CT, MRI, or transthoracic/transesophageal echocardiogram (TTE/TEE), which would show intimal separation with a true and false lumen [2].

The pregnant state places women at a significantly increased risk of common vascular events including venous thromboembolism, myocardial infarction, and stroke, while also increasing the risk of more uncommon vascular events such as aortic dissection and aortic rupture. These rare vascular events can often lead to death of the mother and fetus without prompt treatment [4]. The pregnant state places women at risk due to an increase in circulating blood volume and cardiac output that lead to vascular structural changes. The pathophysiology of aortic dissection in pregnancy is likely related to these known vascular and structural changes leading to degeneration of the aortic media, cystic medial necrosis, and an increased aortic diameter and compliance [5].

Clinical documentation of aortic dissection in pregnancy is limited. As such, there is a lack of clinical trials determining definitive protocols to prevent, screen, and treat pregnant patients with an aortic dissection. This case report aims to describe a patient presenting in the third trimester of pregnancy with an acute diagnosis of a Stanford Type A aortic dissection and review treatment options and outcomes.

## 2. Case

The patient was a 30-year-old G2P1001 at 36 weeks and 4 days who was transferred to Loyola University Medical Center (LUMC) with a Type A aortic dissection. Her pregnancy was complicated by gestational hypertension that had been diagnosed one week prior to her initial presentation. Baseline HELLP labs (which include a CBC, CMP, and urine protein to creatinine ratio) were obtained and were normal at that time. The patient's blood type was O negative and antibody negative and she was Group B streptococcus positive; her serologies were otherwise unremarkable. She had had a prior normal spontaneous vaginal delivery at term without complications. Her past medical history and past surgical history were otherwise noncontributory. She had been taking her prenatal vitamins daily. She was a former smoker having quit one year prior; her social history was otherwise negative for alcohol or recreational drug use. The patient had a negative family history of connective tissue disease or aortic dissection.

**Figure 1:**
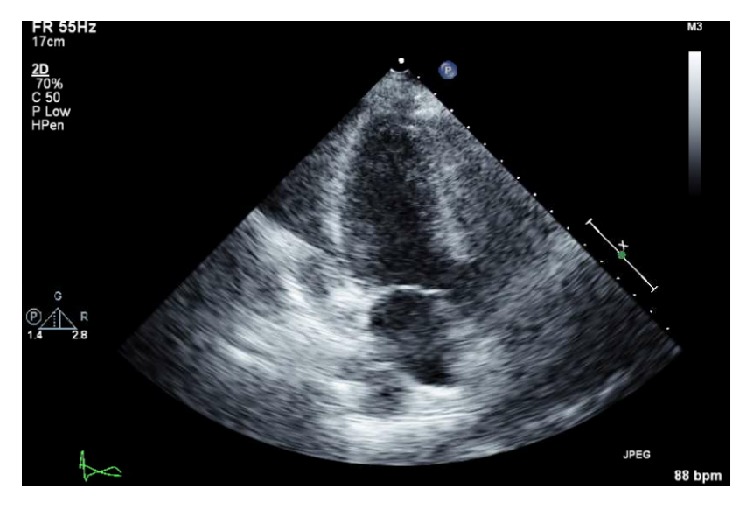
TTE of ascending aorta [6].

She initially presented to an outside hospital two weeks prior to her admission to LUMC, complaining of left-sided chest pain; initial evaluation with EKG and CTPE was negative for acute coronary syndrome and pulmonary embolism and she was discharged home. Over the subsequent two weeks, her chest pain had slowly improved. On the day of her presentation to LUMC, however, her pain acutely increased, characterized as “sharp” and “tearing.” At the outside hospital, CTPE was negative, though the retrospective read of her second CT identified the aortic dissection. EKG showed mild ST-depression and her troponin levels were increased to 1.75. On physical exam, the patient was tachycardic with normal blood pressures and oxygen saturation; she was found to have a new continuous aortic murmur with bounding pulses. She was sent for a TTE with the final read showing evidence of dilation of the ascending aorta (4.2cm) with a dissection flap of aorta seen in the ascending aorta and aortic arch, severe aortic regurgitation, and severe diastolic dysfunction (Figure 1).

She was transferred to our tertiary care center. On presentation to LUMC, her blood pressures were mild range and her HELLP labs and coagulation factors were normal. The fetus had a category I tracing. She was consented for cesarean delivery with repair of aortic dissection. She underwent a primary low transverse cesarean section under general anesthesia; total estimated blood loss (EBL) for this portion of the case was 1L. She was given Pitocin, misoprostol, and a B-lynch was performed for uterine atony. As there was concern for potential coagulopathy related to her aortic repair, a JP drain was left in place. She delivered a liveborn male infant with APGAR score of 9/9 and an umbilical artery pH of 7.34 with a 1+ base excess. After completing the obstetrics portion of the case, the patient underwent an emergency repair of the aortic dissection with resuspension of the aortic valve, primary repair of the left main ostial dissection, insertion of a hemishield graft, and cut-down of the left femoral artery. She was given 4L of cardioplegia, a sterile, isotonic crystalloid solution used to induce cardiac stasis and protect the myocardium during open-heart surgery, and was placed on cardiac bypass during the procedure with a pump time of 230 minutes and a cross-clamp time of 208 minutes [7]. The patient received 500cc of cell saver, 1U of platelets, 2U of FFP, 1U of cryoprecipitate, and 1U of pRBCs; total EBL for the second portion of the case was 800mL. Her postoperative course was uncomplicated, and she was discharged home on postoperative day seven with a hemoglobin of 7.4 and planning to use the copper IUD for contraception.

## 3. Discussion

Aortic dissection in the pregnant population is a relatively uncommon but can be catastrophic for both the mother and fetus. For Type A dissections, mortality increases by 1-3% per hour after presentation, increasing to 25% after 24 hours, 70% by 1 week, and 80% mortality by 2 weeks. Aortic dissection in pregnancy occurs more commonly in the third trimester due to the hyperdynamic state and the hormonal effect on vasculature [8]. In the nonpregnant population, a majority of cases of aortic dissection occur in patients with a previously dilated and therefore weakened aorta; however, in pregnancy, this finding may be absent [6]. Though aortic complications in pregnancy have been reported in women with Marfan syndrome, the vascular type of Ehlers-Danlos syndrome, Turner syndrome, Loeys-Dietz syndrome, and congenital aortic malformations such as bicuspid aortic valve, the majority of cases in the pregnant population occur in women without any other known risk factors [4]. Of note, the only risk factors the presented patient had were pregnancy and a prepregnancy history of tobacco use. Typical for the gravid women with a dissection, the patient presented with nonspecific chest pain. This case highlights the importance of vigilance in evaluating pregnant women with new onset cardiopulmonary symptoms that could be related to one of several life-threatening etiologies.

For Stanford Type A dissections, the mainstay of treatment is surgery whereas, for Type B dissections, treatment involves medical management. Surgical management involves excision of the intimal tear, obliteration of entry into the proximal false lumen, reconstitution of the aorta with interposition of a synthetic vascular graft, and repair or replacement of the aortic valve. Medical management involves beta-blocker administration with the goal to keep blood pressures below 120/80 and endovascular stent grafting for patients with complications related to their dissection. Complications include occlusion of a major aortic branch with end-organ ischemia and persistent severe hypertension or chest pain [7]. When surgically managing pregnant patients beyond 24 weeks' gestation with an aortic dissection, emergent cesarean delivery is recommended before the mother is placed on cardiac bypass. In patients below 24 weeks' gestation, individualized discussions are needed as the risk of fetal demise is high, and delivery does not demonstrate improvement in maternal circulation [8]. Given the high mortality rate of aortic dissections, pregnant patients with and without risk factors should be treated with a high index of suspicion when presenting with characteristic complaints and physical exam findings of aortic dissection.
